# Special Issue “Natural Products as Multitarget Agents in Human Diseases”

**DOI:** 10.3390/ijms27020644

**Published:** 2026-01-08

**Authors:** Andrey S. Marchev, Nikolina M. Mihaylova

**Affiliations:** 1 Laboratory of Eukaryotic Cell Biology, Department of Biotechnology, The Stephan Angeloff Institute of Microbiology, Bulgarian Academy of Sciences, 139 Ruski Blvd., 4000 Plovdiv, Bulgaria; 2 Department of Immunology, The Stephan Angeloff Institute of Microbiology, Bulgarian Academy of Sciences, 1113 Sofia, Bulgaria; mihaylova_n@microbio.bas.bg

For centuries, natural products (NPs), particularly plant-derived phytochemicals, have proven their value as agents with a plethora of health benefits for humans [1,2]. They possess a diverse range of pharmacophores, exhibit stereochemistry, and have metabolite-like properties, while their chemical diversity demonstrates potential for the discovery of novel compounds with unique biological activities, synergy, and complexity [3,4]. Natural products have been investigated as sources for the development of novel therapeutic drugs with enhanced potency that align with sustainability goals. In addition to NPs being validated for traditional medicinal applications, they show promise for use in personalized medicine. The effect of NPs may also be predicted by utilizing contemporary “omics” technologies and bioinformatics [5,6].

Emerging threats to human health require concerted efforts to search for both preventative and treatment strategies, with particular emphasis on phytochemicals in order to establish new therapies and reduce disease spread as well as associated mortality [7,8]. Hence, a quarter of all drugs currently approved by the Food and Drug Administration (FDA) and/or European Medicinal Agency (EMA) are derived from plants [9,10].

Therefore, the aim of this Special Issue is to present novel research findings that allow for an evaluation of the therapeutic effects of NPs, with a focus on the physiological, biochemical, and molecular processes underlying their mechanism of activity, as demonstrated via in vitro and in vivo studies pertaining to human health. In total, five articles and three reviews have been accepted for publication in this Special Issue. Among these studies, there is a focus on revealing the natural therapeutic potential of isorhamnetin in treating severe acute pancreatitis. New insights into alternative and sustainable treatment options for psoriasis based on NP-derived compounds are also provided. Furthermore, the possible prevention and treatment of nonalcoholic fatty liver disease using leonurine is discussed. Some of the involved researchers also study the potential of magniferin and its contribution to treating chronic inflammation and impaired wound healing in diabetes, while others investigate the possible use of veratric acid as a potential therapeutic agent for alleviating hair loss. A number of findings reveal the beneficial effects of cannabinoids in the treatment of type 2 and non-type 2 asthma; others suggest the potential utility of punicalagin and ellagic acid as target therapies for osteoarthritis and demonstrate new opportunities for the management of rheumatoid arthritis, in terms of comprehensive multidisciplinary strategies based on medicinal plants and plant-derived molecules.

The contribution by Li et al. (Contribution 1) reveals the anti-inflammatory properties of isorhamnetin (ISO) based on an in vitro (involving primary pancreatic acinar cells) and in vivo mouse model of severe acute pancreatitis (SAP). The therapeutic promise of ISO was considered to be primarily due to its ability to alleviate pancreatic acinar cell necrosis, as well as its ability to ameliorate mitochondrial dysfunction—particularly by inhibiting mitochondrial ROS generation, preserving ATP production, maintaining mitochondrial membrane potential, and preventing oxidative damage and the release of mitochondrial DNA. It was mechanistically identified that high-temperature requirement A2 (HtrA2) plays a central regulatory role in the protective effect of ISO on mitochondrial dysfunction in sodium taurocholate (STC)-injured acinar cells. Furthermore, an integrated approach involving bioinformatics, molecular docking analysis, and experimental validation indicated that ISO may directly impede the histone demethylation activity of KDM5B, resulting in the restoration of pancreatic HtrA2 expression, and thereby facilitating the preservation of mitochondrial function in pancreatic acinar cells following STC treatment. These discoveries not only present novel insights into the molecular intricacies associated with mitochondrial dysfunction during the progression of SAP, but also open up new avenues for the establishment of therapeutic strategies targeting mitochondrial dysfunction in severe acute biliary pancreatitis.

The research article contributed by Fan et al. (Contribution 2) investigates the critical role of leonurine (a natural product unique to the Lamiaceae plant *Leonurus japonicas* Hout) in the prevention and treatment of nonalcoholic fatty liver disease (NAFLD) in a mouse model. The researchers observed that leonurine altered the gene and lipid profiles of NAFLD in mice, and they preliminarily verified that leonurine had an anti-NAFLD effect, conferred by the phosphorylation of the ADRA1a–AMPK–SCD1 axis. Leonurine intervention reversed the high-fat–high-sugar-diet-induced changes in lipid metabolism-related genes such as stearoyl-CoA desaturase 1 (Scd1), spermine synthase (*Sms*), AP-1 transcription factor subunit (*Fos*), oxysterol binding protein like 5 (*Osbpl5*), and FK506 binding protein 5 (*Fkbp5*) in liver tissues. Liver lipidomic analysis demonstrated that leonurine can alter the abundance of lipid molecules related to fatty acyls (FAs) and glycerophos pholipids (GPs), such as TxB3, carnitine C12-OH, carnitine C18:1-OH, and LPC (20:3/0:0). These finding enrich our understanding of the pathogenesis of NAFLD, contributing to advancements in the establishment of preventative drugs and NAFLD treatments.

Jayasuriya et al. (Contribution 3) present original work focused on investigating the anti-inflammatory potential of magniferin (a natural polyphenol) in managing the inflammatory processes in hyperglycemia-induced macrophages. Mangiferin enhanced nuclear factor erythroid 2-related factor 2 (Nrf2) activation and antioxidant defense in macrophages exposed to hyperglycemic stress, thereby effectively mitigating inflammation and oxidative damage. This activation suppressed nuclear factor kappa-light-chain enhancer of activated B cells (NF-κB) and NLRP3 signaling, diminishing the expression of pro-inflammatory mediators such as cyclooxygenase-2 (COX-2) and interleukin-6 (IL-6). Furthermore, it significantly enhanced the invasiveness and migration of macrophages in a hyperglycemic environment, indicating its potential to improve wound healing. These findings emphasize the potential of mangiferin as a promising therapeutic agent in diabetic wound management.

The effect of veratric acid (VA), a major benzoic acid found in fruits and vegetables, was investigated by You et al., with a focus on its ability to directly activate human hair follicle dermal papilla cells (HFDPCs) and eventually regulate of the pathways involved in hair growth. The results showed that VA promoted the proliferation of HFDPCs and upregulated the growth factors associated with hair growth. Furthermore, VA enhanced ALP activity, facilitated cell aggregation, and increased the expression of key factors involved in follicular inductivity, while simultaneously suppressing apoptosis in HFDPCs. Moreover, VA attenuated both replicative senescence and oxidative stress-induced senescence in HFDPCs by downregulating senescence-associated factors. Taken together, these findings indicate that VA has the potential to mitigate hair loss by promoting HFDPC proliferation and suppressing cellular senescence (Contribution 4).

The review contributed by Lee et al. (Contribution 5) discusses the therapeutic potential of keratinocyte-targeting natural products in psoriasis and highlights their efficacy and safety in comparison with those of conventional treatments, with a particular focus on various related signaling pathways (such as JAK-STAT and NF-κB) and cytokines. The evidence presented in this review highlights the uncovered potential of natural products as sources of novel, sustainable, and potentially safer therapeutic agents for use in the management of psoriasis. Compounds such as luteolin and piperine demonstrated compelling activity in preclinical models, providing evidence of their potential to reduce inflammation, scaling, and the overall severity of psoriatic lesions. Despite this, natural product-derived compounds used for psoriasis therapeutics have demonstrated various limitations, such as low availability, poor solubility, permeability, and stability, diminishing their efficacy when administered orally or topically. For this reason, although natural compounds show anti-psoriatic effects in in vitro or animal models, they may not offer sufficient potency to produce clinically relevant effects in humans. Another limitation is their lack of specificity in that they may end up affecting multiple signaling pathways instead of directly targeting the specific pathways responsible for keratinocyte hyperproliferation in psoriasis. To address these limitations, the authors point to the necessity of developing novel drug delivery systems, combination therapies, and standardized formulations. These advancements must be aimed at improving the bioavailability, specificity, and potency of natural compounds for psoriasis treatment. It is crucial to conduct more rigorous clinical studies to establish the therapeutic potential of these promising natural product-derived compounds.

The review by Lewandowska et al. (Contribution 6) aimed to reveal the beneficial effects of cannabinoids in the treatment of respiratory diseases. Cannabinoids exert certain physiological responses in the respiratory system owing to their immunomodulatory properties and the distinct presence of the endocannabinoid system in the lungs. In animal model studies, ∆9-tetrahydrocannabinol (THC) and cannabidiol (CBD) seemed to counteract bronchoconstriction and inhibit pro-inflammatory mediation. However, THC and CBD should be used with caution, given the alarming data indicating an increased risk of asthma development among recreational cannabis smokers. Furthermore, the potential clinical relevance of THC in counteracting bronchoconstriction, however promising, remains inconclusive and often controversial, particularly in the case of chronic exposure. Perhaps in the future, the adjuvant use of phytocannabinoids in the treatment of asthmatic patients will be justified after individual risk–benefit assessments are conducted. At the moment, further high-quality research on their safest routes of administration and on their long-term health consequences is imperative.

The study by Breland et al. (Contribution 7) provides novel insights into the inhibition of ADAMTS-5,a major metalloprotease involved in regulating the cartilage extracellular matrix, by punicalagin (PCG) and ellagic acid (EA) when used for the treatment of osteoarthritis (OA). Through molecular docking simulations, the authors predicted enzyme–inhibitor binding interactions; the results of said prediction suggest that both compounds may be able to bind within the active site by forming H bonds and interactions between the ligand’s aromatic rings and hydrophobic residue in the enzyme, with inhibition constants of 183.3 µM and 1.13 µM for PCG and EA, respectively, being found. This study also elucidated the specific chemical properties of each ligand that promote and prevent higher binding potential within the enzymatic active site. Subsequent inhibition assays confirmed that both PCG and EA significantly reduced the removal of sulfated glycosaminoglycan (sGAG) from articular cartilage. Furthermore, an examination of the metabolic conversion of PCG into EA in solution with ADAMTS-5 revealed that the enzyme does not play a significant role in accelerating hydrolysis of PCG. These findings suggest that further investigation is warranted to validate the use of that PCG and EA as a prodrug–proactive metabolite pair in OA-targeted therapies and in the development of drug delivery systems targeting arthritic synovial joints.

The review by Nikolova-Ganeva et al. (Contribution 8) presents not only the basic features of rheumatoid arthritis (RA) pathogenesis, its risk factors, and the involved signaling pathways, but also underscores the progress made through research using pre-clinical RA in in vitro and in vivo models; this progress has opened up new avenues for the management of this disease, particularly comprehensive multidisciplinary strategies based on medicinal plants and plant-derived molecules. Although there are a number of drugs and novel biological therapies available, they present several limitations and drawbacks, including the fact that they do not always prevent disease recurrence and may cause adverse effects after long-term use. Hence, it is still necessary to unveil the cause(s) of RA in order to ensure that it can be cured or at least prevented. Novel therapies need to be established and the diverse yet unidentified signaling pathways in non-responders need to be specified. To increase the efficiency of plant-derived extract or molecules, these may be included into novel delivery systems, such as nanoparticles, which could improve their pharmacological and therapeutic properties. Their encapsulation allows for precise dosage, the prolonged and controlled release of phytochemicals, and reduced dosage regimens and drug toxicity, improving patient compliance. Using different “omics” technologies, such as metabolomics, 2D and 3D in vitro models, and different panels of in vivo models, may also help us overcome some of the challenges associated with using plant-derived products; these technologies may finally reduce the likelihood of failure in clinical trials on potential new therapies. Plant-derived products also contain prebiotic components, the interactions of which with the host microbiome might have a significant impact on health and disease. A future task for researchers in this field will be to identify how these parameters interact to trigger an autoimmune inflammatory response and the development of Ra; this would further help to optimize the selection of plant-derived products for therapy and define their mechanisms of activity. In the near future, additional research efforts might be invested in combining phytotherapy with stem cell research, clustered regularly interspaced short palindromic repeats, and CRISPR-associated protein 9 (CRISPR-Cas9) genome editing or gene therapy, which would provide a long-term therapeutic advantage for RA patients, following the evaluation of safety, ethical, and medical concerns.
